# Relationship between 2-Hour Tacrolimus Concentrations and Clinical Outcomes in Long Term Kidney Transplantation

**DOI:** 10.3390/pharmacy8020060

**Published:** 2020-04-03

**Authors:** Jeffrey Yin, Tammy Hsu, Janice S Kerr, Robert Steiner, Linda Awdishu

**Affiliations:** 1 Department of Pharmacy, UC San Diego Health System, 200 West Arbor Dr., San Diego, CA 92103, USA; jskerr@health.ucsd.edu; 2 Division of Clinical Pharmacy, UC San Diego Skaggs School of Pharmacy and Pharmaceutical Sciences, La Jolla, CA 92093, USA; tammyhsu@mednet.ucla.edu (T.H.); lawdishu@health.ucsd.edu (L.A.); 3 Division of Nephrology, Department of Medicine, UC San Diego School of Medicine, La Jolla, CA 92093, USA; rsteiner@health.ucsd.edu

**Keywords:** tacrolimus, kidney transplantation, drug monitoring

## Abstract

**Background:** Tacrolimus is routinely monitored using trough concentrations, however, recent data have suggested that area under the curve (AUC) provides better correlation with toxicity and efficacy. Area under the curve is cumbersome to measure, but studies have demonstrated that surrogate time points such as 2-hour concentrations are well correlated with AUC. **Methods:** This is a single center, retrospective study of adult kidney transplant recipients with 2-hour tacrolimus concentrations measured over three years post-transplant. The primary outcome was to determine the difference in serum creatinine (Scr) in those with 2-hour tacrolimus concentrations greater than 20 ng/mL versus those less than or equal to 20 ng/mL. **Results:** A total of 150 kidney transplant recipients were included. The mean Scr and glomerular filtration rate were 1.49 ± 1.01 mg/dL and 59 ± 23.2 mL/min/1.73 m^2^, respectively, for the entire cohort. The rate of donor specific antibody formation was 2% and 8% experienced biopsy-proven rejection. The rate of cytomegalovirus viremia was 2% and BK viremia was 13%. There was no significant difference in kidney function over 36 months for the groups specified a priori. **Conclusions:** Long-term outcomes of maintaining tacrolimus 2-hour concentrations over 20 ng/mL is favorable with minimal opportunistic infections.

## 1. Introduction

Tacrolimus (FK) is a calcineurin inhibitor most commonly used in immunosuppressive regimens after kidney transplantation [1]. It has a narrow therapeutic index with high inter- and intra-individual variability, necessitating the need for therapeutic drug monitoring [2,3]. Traditionally, 12-hour whole blood trough concentrations are measured and monitored as surrogate indicators of efficacy for the immediate-release version of tacrolimus [2,4,5,6]. However, rejection and toxicity (such as nephrotoxicity, neurotoxicity, diabetes, and opportunistic infections) have been observed within the same concentration range [3,7,8,9], prompting scrutiny on the appropriateness of measuring trough concentrations as a surrogate for both efficacy and toxicity. Studies have demonstrated that while trough concentrations may correlate with toxicity [8,10], area under the curve (AUC) is a stronger predictor of acute rejection [11,12,13]. However, AUC determination requires intense sampling with six or more concentration-time points, limiting its utility for routine monitoring. Studies have demonstrated 2-hour tacrolimus concentrations (C2) correlating with AUC and may be used for monitoring [9,14,15,16].

At our institution, we have implemented routine trough and C2 therapeutic drug monitoring for immediate-release tacrolimus. The bioavailability of tacrolimus is low, ranging from 17–23% [17]. Since food reduces the absorption of tacrolimus, decreasing AUC by approximately 28–37% [17], patients are instructed to take the dose one hour before meals or three hours after meals to maximize absorption. The purpose of this study was to review the outcomes of trough and C2 monitoring for kidney transplant recipients receiving immediate-release tacrolimus. Based on manufacturer pharmacokinetic studies of tacrolimus in kidney transplant patients, tacrolimus was found to have a mean maximum concentration (Cmax) of at least 19.2 ± 10.3 ng/mL for patients with observed troughs ranging from 7–20 ng/mL [17]. We therefore hypothesize that patients receiving immediate-release tacrolimus with C2 > 20 ng/mL will have a greater AUC than those with C2 ≤ 20 ng/mL, and have improved graft function and decreased incidence of rejection without an increase in the incidence of opportunistic infections. 

## 2. Materials and Methods

This is a single center, retrospective, observational cohort study of adult kidney transplant recipients at the University of California San Diego Health System between 2009 and 2012. Patients met the inclusion criteria if they were 18 years or older, kidney transplant recipients, and were receiving immediate-release tacrolimus for immunosuppression. Patients were excluded from this study if they were multi-organ transplant recipients, re-transplant recipients, receiving cyclosporine, sirolimus, or employing calcineurin minimization strategies. This study was approved by the institutional review board on 16 September 2014 for project #140932X. 

The standard of care at UC San Diego Health System is for all patients to receive thymoglobulin induction, unless patients have concurrent hepatitis C (HCV) or human immunodeficiency virus (HIV) infection. Patients are stratified into the high risk group as opposed to the low risk group if the patient had any of the following criteria: panel reactive antibodies (PRA) ≥20%, positive donor specific antibodies (DSA), if kidneys are from donors after cardiac death (DCD), if delayed graft function (DGF) or slow graft function (SGF) is observed, or if the patient is African American. Patients are initiated on immediate-release tacrolimus at 0.1 mg/kg/day, which is administered in a fasting state, starting on post-operative day 0 up to day 3, depending on their risk level. Patients had a target trough of 10–13 ng/mL from days 0–45, tapered down to 8–10 ng/mL thereafter. If patients were deemed to be high risk, a higher trough target of 13–16 ng/mL was used from days 0–45, tapered down to 10–13 ng/mL from days 45–90, then further tapered down to 8–10 ng/mL thereafter. The in-house tacrolimus assay used during the time of the study was a chemiluminescent micro particle immunoassay.

Methylprednisolone is initiated during surgery, and gradually tapered, as described in our protocol. Mycophenolate is initiated prior to discharge. Cytomegalovirus (CMV) risk is stratified based on the donor and recipient CMV serostatus and type of induction agent used. Valganciclovir prophylaxis is prescribed for six months if high risk, three months if moderate risk, and acyclovir is prescribed for three months if low risk. Surveillance for CMV includes monitoring polymerase chain reaction (PCR) every 3–6 months thereafter as needed. BK viremia is monitored monthly until three months and then on an as needed basis. 

Patients are counseled and educated on the importance of taking tacrolimus on an empty stomach as well as the need for them to have both a tacrolimus trough and C2 drawn on the same day. Laboratory staff are trained to draw the tacrolimus trough, and advise patients to return for the tacrolimus C2 90–120 minutes after they have taken their dose. 

The following variables were collected at baseline (at time of discharge from transplant admission): age, gender, race, date of transplant, type of transplant (living or deceased donor), comorbidities prior to transplantation, indication for kidney transplant, and type of induction therapy received at time of transplant. Additional variables were collected at baseline and at six month intervals up to 36 months: weight, serum creatinine (Scr), urine protein to creatinine ratio (UPC), BK PCR, CMV PCR, tacrolimus daily dose, trough concentrations, C2, prednisone (Pred) dose, mycophenolate (MMF) dose, and whether the patient was receiving any cytochrome P450 inhibitors or inducers (e.g., diltiazem, azole antifungals, phenytoin, carbamazepine). If a trough and/or C2 concentration was not available at the exact time point, the closest trough and/or C2 concentration around that time point was used. If none were available, then the data point was omitted. If the patient underwent a biopsy (i.e., for cause biopsy), the biopsy date, biopsy result, Scr, tacrolimus dose, trough concentration, and C2 prior to biopsy were collected. If DSA was measured, the date and result were collected. Glomerular filtration rate (GFR) was estimated using the CKD-EPI Creatinine Equation [18]. The primary outcome was Scr values at 6-month intervals up to 36 months. Secondary outcomes included GFR, incidence of BK viremia, CMV viremia, DSA, and biopsy proven rejection at 6-month intervals up to 36 months. 

For the primary analysis, the Scr was compared between patients with tacrolimus C2 greater than 20 ng/mL and those with tacrolimus C2 less than or equal to 20 ng/mL. Based on a previous study from our institution, the average Scr value at one-year post transplant was 1.48 ± 0.49 mg/dL [19]. In order to find a difference in Scr of 20%, which correlated to a change of 0.3 mg/dL ± 0.49 mg/dL, assuming an alpha of 0.05, we would need 43 patients in each group to achieve an 80% power. For the secondary analysis, incidence of BK viremia, CMV viremia, DSA, and biopsy-proven rejection were compared between the two groups. The Mann–Whitney-U test was used to compare Scr and GFR, while Fisher’s exact test was used to compare the incidence of BK viremia, CMV viremia, DSA, and biopsy-proven rejection. Data were extracted electronically from the electronic medical record when feasible and recorded in Microsoft Excel (2010) and statistical analysis was performed using R software (version 2.15.3). 

## 3. Results

**Figure 1:**
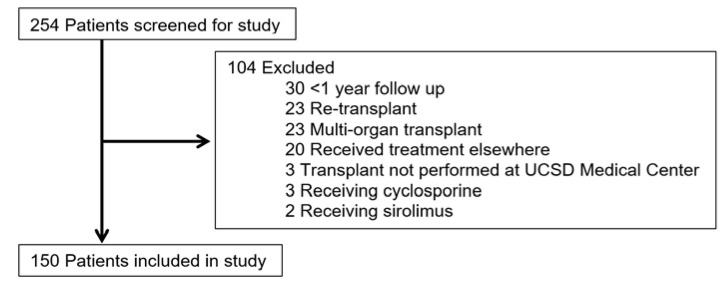
Patient disposition: the flow of eligible patients and reasons for exclusion from the study.

During the study period, a total of 254 patients were screened, and 150 patients met the inclusion criteria with approximately 50% of subjects having follow up at three years (Figure 1).

Baseline demographics are shown in Table 1. The subjects were 49.8 years on average, 60% male, and diabetes was the most common cause of end stage renal disease prior to transplantation. The majority received deceased donor kidney transplant, thymoglobulin induction, and were started on triple immunosuppression therapy (tacrolimus, mycophenolate, and prednisone) by the time of discharge.

A summary of the immunosuppression regimen is listed in Table 2. Kidney function in the entire cohort, as estimated by GFR, increased at six months relative to the baseline then remained relatively stable with an increase at 36 months. There was a decline in tacrolimus dose per day, tacrolimus dose per kilogram per day, and prednisone dose from baseline to three years, with the greatest change occurring between baseline and six months, which is in agreement with our center-specific protocol. The tacrolimus trough and mycophenolate dose exhibited less decline, with minor fluctuations in tacrolimus average trough concentrations between 24 to 36 months. The number of patients on triple immunosuppression therapy decreased between baseline and 18 months, then remained relatively stable from 24 through to 36 months. This was due to stopping mycophenolate for adverse effects. The average dose change after 12 months was essentially unchanged through 36 months, with equal percentages of increases versus decreases in tacrolimus dose. The percentage of patients with a change in tacrolimus dose greater than 30% was 19–41% at each time point from 12 months onward, reflecting stable immunosuppression. 

**Figure 2:**
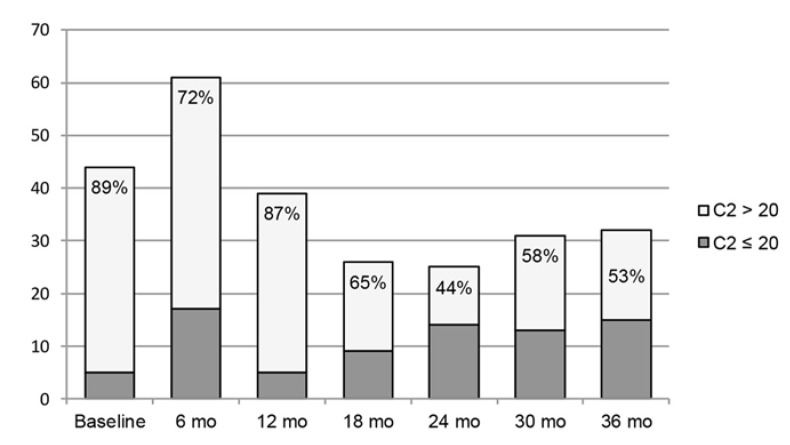
Number of patients with 2-hour tacrolimus concentrations drawn: figure above depicts the number of patients with 2-hour tacrolimus concentrations at six month intervals up to 36 months as well as the proportion of patients with C2 levels > 20 ng/mL versus those with C2 levels ≤ 20 ng/mL. Abbreviations used: mo = months, C2 = 2-hour tacrolimus concentration.

Over the first 12 months, the majority of C2 concentrations were greater than 20 ng/mL (72–89%, Figure 2). After the 12-month period, C2 concentrations above 20 ng/mL declined, but tended to be more frequent than C2 concentrations less than 20ng/mL, with the exception of the 24-month time point (53–65%, Figure 2). 

**Figure 3:**
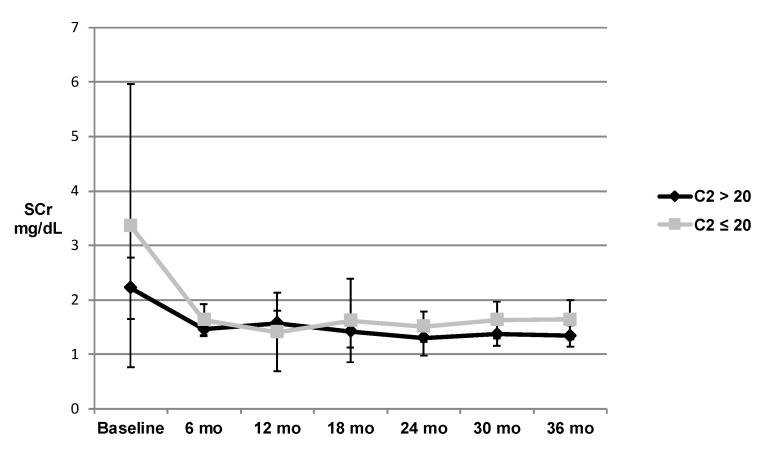
SCr in patients with 2-hour tacrolimus concentrations >20 vs. ≤20 ng/mL: figure above depicts SCr of patients with C2 levels >20 ng/mL versus those with C2 levels ≤20 ng/mL as well as the 95% confidence interval for the SCr of each group at six month intervals up to 36 months. Abbreviations used: SCr = serum creatinine, mo = months, C2 = 2-hour tacrolimus concentration.

**Figure 4:**
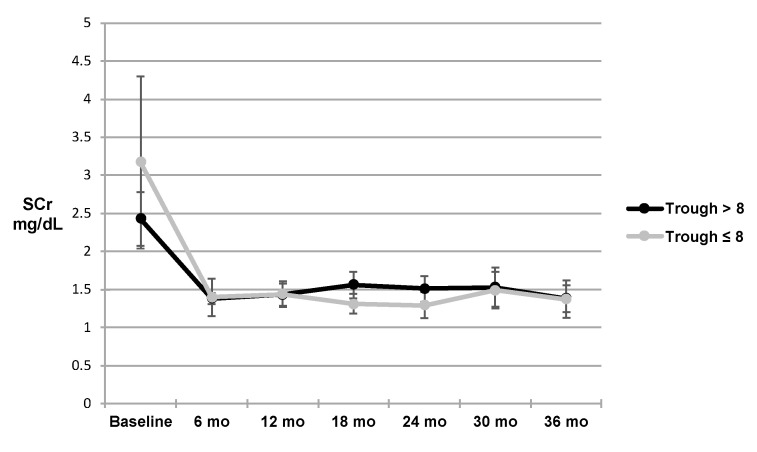
SCr in patients with trough concentrations >8 vs. ≤8 ng/mL: figure above depicts SCr of patients with trough levels >8 ng/mL versus those with trough concentrations ≤8 ng/mL as well as the 95% confidence intervals for the SCr of each group at six month intervals up to 36 months. Abbreviations used: SCr = serum creatinine, mo = months.

**Figure 5:**
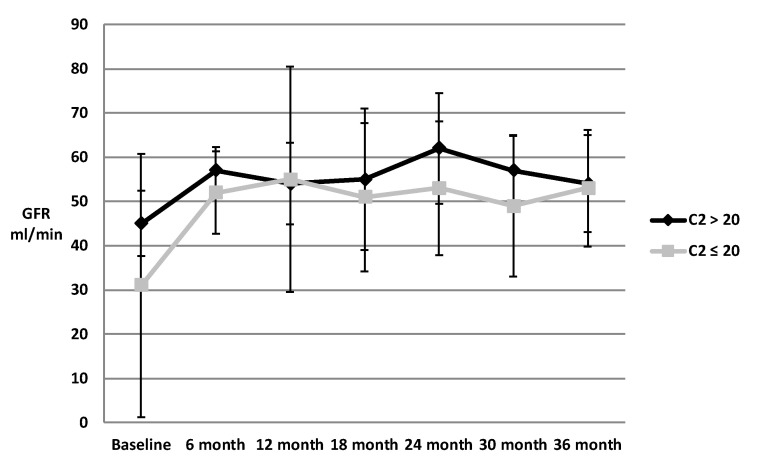
GFR in patients with 2-hour tacrolimus concentrations >20 vs. ≤20 ng/mL: figure above depicts GFR of patients with C2 > 20 ng/mL versus those with C2 ≤ 20 ng/mL as well as the 95% confidence intervals for the GFR of each group at six month intervals up to 36 months. Abbreviations used: C2 = 2-hour tacrolimus concentration, GFR = glomerular filtration rate, mo = months.

Kidney function in terms of SCr for the respective tacrolimus C2 groups are shown in Figure 3. No statistically significant difference was found between the higher and lower exposure groups. However, fewer patients had a C2 measured at the later time points. There was no clear trend after baseline when grouping the patients by trough concentrations, as shown in Figure 4. Kidney function expressed in terms of GFR for the respective tacrolimus C2 groups are shown in Figure 5. There was also no statistically significant difference, though the C2 > 20 ng/mL group tended to have slightly higher GFR at all time points except for at 12 months and at 36 months. Of note, 40 patients were reclassified at least once from their original C2 concentration group (i.e., initially were in the C2 concentration greater than 20 ng/mL group, but at a different time point fell in the C2 concentration less than or equal to 20 ng/mL group, and vice versa).

The overall rate of DSA formation was low and is summarized in Table 3. A total of 71 subjects had DSAs measured and three positive DSAs were noted. A total of 26 biopsies were performed, with 13 confirming rejection. Of those with biopsy-proven rejection, four had C2 concentrations greater than 20 ng/mL around the time of biopsy, while three had C2 concentrations less than or equal to 20 ng/mL. The remaining six did not have C2 concentrations drawn around the time of biopsy.

A summary of the opportunistic infections is listed in Table 4. The number of subjects with CMV infection was low, with one positive infection between six and 12 months, and one by 36 months. De novo cases of BK viremia were highest in the first six months, gradually decreasing over time.

## 4. Discussion

The primary objective of our study was to determine if patients receiving higher levels of immunosuppression as defined by a tacrolimus C2 greater than 20 ng/mL had improvements in kidney function over a 3-year period. We were not able to detect a statistically significant difference in kidney function between the groups. However, we found overall that our cohort of patients had a low rate of antibody formation, acute rejection, and incidence of CMV viremia, while the incidence of BK viremia was similar to the national averages. The 3-year graft function was good, as measured by an average SCr of 1.49 mg/dL and GFR of 59 mL/min/1.73 m^2^ with a rate of decline of approximately 1 mL/min/1.73 m^2^ per year.

A higher proportion of patients had C2 concentrations >20 ng/mL, reflecting adequate absorption likely a result of fasting administration of tacrolimus. Studies have found that co-administration of tacrolimus with food can result in a decrease in Cmax ranging from 65–77%. [17] Tacrolimus dose requirements decreased over time, which can be explained in part from lower long-term target trough concentrations, but also possibly from findings that have previously been described such as time dependent changes in tacrolimus clearance [20] and/or oral bioavailability [14]. Reductions in dose resulted in small changes in average trough concentrations with larger fluctuations in C2. Due to fluctuations in the ratio of tacrolimus peak (C2)/trough concentrations (between two to three), we feel that this parameter should not be used as a correlate of AUC. 

Although early pharmacokinetic studies of tacrolimus suggested that trough concentrations may be predictive of toxicity and efficacy [2,4,5,6], later studies have found that trough concentrations correlate with toxicity, but not efficacy [8,10]. As AUC has been found to be the best correlate with efficacy, numerous studies have attempted to find single time point correlates of AUC in order to provide a practical approach to monitor AUC [9,14,15,16]. Although results have been conflicting, there is growing consensus that trough concentrations may not provide the best correlation with AUC [21,22]. Additionally, the pharmacokinetics of tacrolimus have been found to change over time, further arguing the utility of single time point monitoring [9,20,22]. Studies have demonstrated that two-point sampling strategies including a trough and a peak concentration correlate better with AUC, with C2 having one of the better correlations [14]. However, most studies are limited due to small sample sizes, short durations of follow up, and to our knowledge, none have correlated with long-term clinical outcomes.

The national average incidence of rejection post-transplant is approximately 10% at 12 months and 16–18% at 36 months, depending on whether the kidney transplant was from a deceased or live donor [23,24]. Our rates of rejection at 12 months and 36 months were 6% and 8%, respectively, which is lower than the national average. Our results show that the mean time to first rejection is 14 months, with 69% of biopsy-confirmed rejection occurring within the first year. 

Improved tacrolimus exposure through increased AUC/drug levels might increase incidence of calcineurin-inhibitor (CNI) toxicity and opportunistic infections. National average rates of CNI toxicity are difficult to compare, as most studies assessed CNI toxicity rates of both cyclosporine and tacrolimus combined. We documented a low rate of CNI toxicity, 3.8%, for subjects who had for cause kidney biopsies. 

The incidence of CMV viremia was shown to be approximately 5.8% in kidney transplant patients with a mean follow up of 33.6 months in one study [25], and approximately 7% in another study followed over 24 months [26]. The incidence of CMV was 2% over 36 months at our institution. Similarly, our rate of BK viremia was comparable to studies documenting rates of 11.5–13.7% in the first year after transplant [27,28], and overall rates of 10–15% [29].

Although the addition of C2 monitoring may have slight increases in cost and complexity for patients, based on our results, it allows for increased immunosuppression without added risk of CNI toxicity or opportunistic infection, suggesting the utility of such a monitoring strategy.

There are several limitations of this study. The study was retrospective and observational in nature, describing standard of care treatment. Patients may have obtained their C2 or trough level at the incorrect time point, though our patients and laboratory staff have been well trained to minimize this source of error. The number of C2 concentrations reported for each patient was less than expected with attrition leading to loss of power to detect a difference in kidney function. Additionally, the C2 concentration of 20 ng/mL was used based on prior pharmacokinetic studies, suggesting a peak to trough ratio of 2, however, we did not specifically evaluate other C2 concentrations as measures of efficacy. Immunosuppression was individualized in this study. Measurement of C2 concentrations and DSA as well as the decision to perform a kidney biopsy were done for cause rather than per protocol. Our findings are biased, since higher risk patients had more interventions made. Contamination bias may have been introduced if patients switched between immunosuppression profiles at different time points (i.e., one patient was in the C2 > 20 ng/mL group at one time point, but was found to be in the C2 ≤ 20 ng/mL group at another time point). Laboratory measurement errors from interferences and lot-to-lot variability of the immunoassay used may also affect the accuracy of the measurement. Finally, attrition of patients may have introduced bias in either direction, data may be reflective of survivors or adherent patients or alternatively, subjects at greater risk who were followed more closely. 

Of note, the results found in this study apply to the immediate-release version of tacrolimus only. There are currently two other formulations of tacrolimus on the market, tacrolimus extended-release capsules (Astagraf XL, Astellas Pharma US, Northbrook, IL) and tacrolimus extended-release tablets (Envarsus XR, Veloxis Pharmaceuticals, Cary, NC), also known as LCP-Tacro [30,31,32]. Their pharmacokinetics, especially the LCP-Tacro, are quite different from tacrolimus immediate-release, thus, data are needed to confirm if C2 monitoring also provides improved efficacy and correlation with AUC. Of interest, food also decreases absorption for these two extended-release versions of tacrolimus, with LCP-Tacro having the most impact with a decrease in AUC up to 55% [23]. The manufacturer of both extended-release versions also recommends an empty stomach protocol, similar to the one used at our institution [22,23].

Further studies are warranted to examine the relationship between monitoring tacrolimus using combined C2 and trough concentrations and graft outcomes in larger samples and additional transplant populations.

## 5. Conclusions

Tacrolimus 2-hour concentrations greater than 20 ng/mL were not associated with an improvement in long-term graft outcomes. However, many patients were able to achieve these 2-hour concentrations without increased risk of CNI toxicity or opportunistic infection. Further research is required to determine the appropriate target 2-hour concentration to optimize long term graft outcomes.

## Figures and Tables

**Table 1:** Baseline demographics for patients eligible to be included in the study including comorbidities and immunosuppression regimen.

Variable	Mean ± SD, N (%)
Age (years)	49.8 ± 13.2
Gender	
Male	90 (60)
Weight (kg)	75.8 ± 17.4
BMI (kg/m^2^)	26.9 ± 4.9
CKD Etiology	
Glomerulonephritis	47 (31)
Diabetes	52 (35)
Polycystic kidney disease	15 (10)
Other	36 (24)
Pre-transplant Comorbidities	
Diabetes	72 (48)
Hypertension	145 (97)
Pre-transplant Scr (mg/dL)	7.67 ± 3.14
Type of transplant	
Deceased	101 (67)
Living Related	31 (21)
Living Unrelated	18 (12)
Induction	
Basiliximab	2 (1)
Thymoglobulin	125 (84)
None	23 (15)
Triple Immunosuppression (FK+ MMF+ Pred)	141 (94)

Abbreviations: SD = standard deviation, BMI = body mass index, CKD = chronic kidney disease, SCr = serum creatinine, FK = tacrolimus, MMF = mycophenolate, Pred = prednisone.

**Table 2:** Summary of immunosuppression and renal function: table below depicts immunosuppression regimen, SCr, and GFR as well as dose changes relative to the dose at 12 months at six month intervals up to 36 months.

Variable (mean)	Baseline N = 150	6 Months N = 150	12 Months N = 126	18 Months N = 107	24 Months N = 83	30 Months N = 74	36 Months N = 63
SCr (mg/dL)	2.54	1.39	1.45	1.51	1.53	1.6	1.49
GFR (mL/min)	43	59	58	57	56	55	59
FK dose (mg/day)	15.46	8.67	7.43	6.58	6.02	6.09	5.61
FK dose (mg/kg/day)	0.043	0.024	0.020	0.018	0.018	0.016	0.015
FK C2 (ng/mL)	34.55	27.67	30.84	26.74	19.45	23.32	21.71
FK trough (ng/mL)	11.10	10.85	10.06	9.08	8.75	9.31	9.14
FK C2/trough	3.11	2.55	3.07	2.94	2.22	2.50	2.38
Pred dose (mg/day)	31.23	7.83	7.24	7.09	7.22	7.17	7.02
Pred dose (mg/kg/day)	0.41	0.1	0.09	0.09	0.09	0.09	0.09
MMF dose (mg/day)	1385.3	1063	915.7	944.4	862.7	894.4	853.7
MMF dose (mg/kg/day)	18.27	14.09	11.94	12.19	11.22	11.36	10.68
% Triple Immunosuppression	94	71	68	67	60	61	65
Average % dose change relative to 12 month	N/A	N/A	N/A	0.99	0.99	0.96	0.99
# of patients with > 30% dose change	N/A	N/A	N/A	20	21	23	26

Abbreviations used: SCr = serum creatinine, GFR = glomerular filtration rate, FK = tacrolimus, C2 = 2-hour tacrolimus concentration, Pred = prednisone, MMF = mycophenolate.

**Table 3:** Clinical outcomes at time of kidney biopsy: table below depicts results of biopsies, number of samples that were associated with DSA, immunosuppression at time of biopsy, and time to biopsy-proven rejection relative to transplantation.

Variable	Mean ± SD, N (%)
Biopsy	26 (17.3)
Biopsy Results	
Cellular Rejection	13
Acute tubular necrosis	4
Calcineurin inhibitor toxicity	1
Membranoproliferative glomerulonephritis	1
Donor specific antibody detected	3 (2)
SCr at biopsy (mg/dL)	2.57 ± 1.48
FK dose (mg/day)	12.08 ± 6.77
FK C2 (ng/mL)	26.28 ± 6.73
FK trough (ng/mL)	10.73 ± 3.76
Median time to first rejection	1.2 years

Abbreviations used: DSA = donor specific antibody, SD = standard deviation, SCr = serum creatinine, FK = tacrolimus, C2 = 2-hour tacrolimus concentration.

**Table 4:** Opportunistic infections: table below depicts number of de novo cases of CMV and BK viremia at 6 months intervals up to 36 months. Abbreviations used: CMV = cytomegalovirus.

Variable	6 Months	12 Months	18 Months	24 Months	30 Months	36 Months
CMV, N (%)	1 (0.7)	1 (0.8)	0 (0)	0 (0)	0 (0)	1 (1.6)
BK, N (%)	14 (10.2)	3 (3.2)	2 (3.1)	0 (0)	1 (2.2)	0 (0)
